# Using the Nine Common Themes of Good Practice checklist as a tool for evaluating the research priority setting process of a provincial research and program evaluation program

**DOI:** 10.1186/s12961-016-0092-5

**Published:** 2016-03-23

**Authors:** Rebecca L. Mador, Kathy Kornas, Anne Simard, Vinita Haroun

**Affiliations:** Public Health Ontario, Santé publique Ontario, 480 University Avenue, Suite 300, Toronto, ON M5G 1V2 Canada; Ontario Long-Term Care Association, 425 University Avenue, Suite 500, Toronto, ON M5G 1T6 Canada

**Keywords:** Evaluation, Priority setting, Public health, Research priorities

## Abstract

**Background:**

Given the context-specific nature of health research prioritization and the obligation to effectively allocate resources to initiatives that will achieve the greatest impact, evaluation of priority setting processes can refine and strengthen such exercises and their outcomes. However, guidance is needed on evaluation tools that can be applied to research priority setting. This paper describes the adaption and application of a conceptual framework to evaluate a research priority setting exercise operating within the public health sector in Ontario, Canada.

**Methods:**

The Nine Common Themes of Good Practice checklist, described by Viergever et al. (Health Res Policy Syst 8:36, 2010) was used as the conceptual framework to evaluate the research priority setting process developed for the Locally Driven Collaborative Projects (LDCP) program in Ontario, Canada. Multiple data sources were used to inform the evaluation, including a review of selected priority setting approaches, surveys with priority setting participants, document review, and consultation with the program advisory committee.

**Results:**

The evaluation assisted in identifying improvements to six elements of the LDCP priority setting process. The modifications were aimed at improving inclusiveness, information gathering practices, planning for project implementation, and evaluation. In addition, the findings identified that the timing of priority setting activities and level of control over the process were key factors that influenced the ability to effectively implement changes.

**Conclusions:**

The findings demonstrate the novel adaptation and application of the ‘Nine Common Themes of Good Practice checklist’ as a tool for evaluating a research priority setting exercise. The tool can guide the development of evaluation questions and enables the assessment of key constructs related to the design and delivery of a research priority setting process.

**Electronic supplementary material:**

The online version of this article (doi:10.1186/s12961-016-0092-5) contains supplementary material, which is available to authorized users.

## Background

Health research priority setting seeks to select priorities that will have the largest benefit to the health of populations, reduce duplication of effort and promote collaboration [1]. Identifying research priorities that will have the greatest impact on policy or practice is critically important. Yet, the process of achieving consensus is often a complex and difficult one [2]. A myriad of methodological approaches to designing and implementing research priority setting processes have been published for a variety of contexts (e.g. [3–10]). Given that there is no gold standard approach to research prioritization [11], there is a need to understand the strengths and weaknesses of different models and their effectiveness in achieving desired outcomes [12]. However, to date, published evaluations of priority setting exercises are lacking [8].

Bryant et al. [12] emphasized this limitation in a narrative review of health research priority setting methods, models and frameworks used in high-income countries and found that, among the 11 different priority setting exercises identified, none had been evaluated to assess the process employed or the extent to which the exercise had achieved its goals. The lack of evaluation is consistent with priority setting exercises conducted in low- and middle-income countries [8]. Evaluating the process and outcomes of a research priority setting exercise is necessary if improvements are to be identified in a way that is systematic, evidence-informed and transparent. However, there is limited guidance on how these evaluations should be conducted, what questions should guide the assessment, and the types of indicators that could be used to measure priority setting constructs [13].

This paper seeks to build on the discussion about evidence-informed priority setting processes by describing the adaption and application of a conceptual framework to the evaluation of a research priority setting process that occurs within the public health sector in Ontario, Canada. Specifically, we describe how we used Viergever et al.’s ‘Nine Common Themes of Good Practice’ checklist [11] (the Checklist) to develop focused evaluation questions, identify indicators, interpret data, and shape recommendations. Although the checklist was designed to facilitate the planning and implementation of a research priority setting process, we describe its utility as an evaluation tool. This paper seeks to provide guidance to others who are embarking on an assessment of a research priority setting process by showing how this framework can be used to design a robust evaluation that identifies recommendations to better align priority setting activities with the overarching goals of the process.

### Overview of the locally-driven collaborative projects (LDCP) priority setting process

LDCP is a program delivered by Public Health Ontario (PHO) – a crown corporation dedicated to protecting and promoting the health of Ontarians and reducing health inequities. The LDCP program’s goal is to support Ontario Public Health Units (PHUs) to work collaboratively on applied research and program evaluation projects related to critical public health problems [14]. Operating on a 2-year cycle, the LDCP program begins with a facilitated process that enables Ontario’s 36 PHUs to collaboratively identify and prioritize ideas for research projects that are relevant to their needs. Once the priority setting process is completed, collaborative teams are established and each LDCP team refines their prioritized research question, develops objectives, and creates a research protocol. In addition to facilitating the prioritization of research ideas and development of protocols, PHO provides funding to support project implementation as well as the team’s knowledge translation activities [15].

The LDCP priority setting process has three overarching goals: (1) to ensure that the process is driven by Ontario PHUs; (2) to prioritize research questions that are relevant to the needs and priorities of PHUs; and (3) to engage a broad cross-section of PHU staff at different phases in the prioritization process. To meet these goals, a two-phased approach was developed and implemented over the course of approximately 3 months in 2012 (Table 1).

**Table 1:** Process used in the 2012 cycle to identify research priorities for locally-driven collaborative projects (LDCPs)

Phase 1: Survey to prioritize subject areas		
Health units submitted a survey to the LDCP program identifying five subject areas of greatest interest. Interest was calculated by summing the total number of health units that selected each subject area. The top seven subject areas with greatest interest moved forward to phase 2.		
Phase 2: Workshop to prioritize 1 research question in each subject area		
Step 1	Formulating potential research questions • Participants provided with an opportunity to formulate research questions of interest to their health unit within the prioritized subject area and topics • Participants shared questions generated by each group member	Number of research questions at the end of the step that move forward for further prioritization
		>20
Step 2	Narrowing down potential research questions • Using a consensus-based decision-making process, research questions were refined and reduced • Six principles guided discussion and decision-making: duplications, already done, misalignment, out-of-scope, too big, too early • Approximately 12 research questions move forward to Step 2.3 – applying criteria of ‘interest’ and ‘impact’	~12
Step 3	Applying criteria of ‘interest’ and ‘impact’ • Participants were asked to consider criteria of interest and vote for three research questions that best met this criteria • Top five questions with potential for greatest impact were ranked and moved forward to Step 2.4 – considering criteria of ‘balance’	5
Step 4	Considering criteria of ‘balance’ • Participants engaged in a discussion about criteria of balance as it relates to the three remaining potential research questions • Comments captured on flip-chart paper • All five questions move forward to Step 2.5 – applying criteria of interest, impact, and balance to identify top research question	5
Step 5	Applying criteria of interest, impact and balance to identify top research question • Participants were asked to begin by considering criteria of interest and vote for one research question that their organization would be most interested in • Participants were next asked to consider criteria of impact and vote for one research question that relates to the most important public health issue • Finally, participants asked to consider the criteria of balance and vote for one research question that will have the most significant benefit on the public health system as a whole • The research question with the most votes moved forward to be collaboratively developed into an LDCP	1

### Phase 1 – Survey

The first phase of the LDCP research prioritization process involved each of the 36 PHUs identifying subject areas most closely aligned with their organization’s needs and priorities. Potential subject areas were firmly rooted in the work that PHUs do, drawn from the Ontario Public Health Program Standards, and the legislated requirements for local public health programs and services [16]. One survey response was requested per PHU, via an email sent to the Medial Officer of Health. Each PHU determined how best to provide a collective response and how to engage staff within the organization. Surveys were collected electronically, results tallied and the seven subject areas that received the most votes were communicated back to the sector as the collective priorities.

### Phase 2 – Workshop

**Fig. 1:**
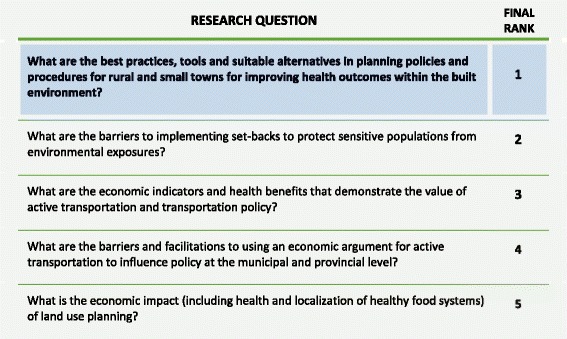
Prioritized research or evaluation questions in the built environment subject area

The second phase was an all-day workshop involving representatives from each of the 36 PHUs. Representatives worked in small groups aligned with each of the seven prioritized subject areas (Table 1). Using a nominal group technique, facilitators guided workshop participants as they articulated potential research questions, refined questions and reduced duplicates, and used a set of pre-defined criteria (Table 2) to rank research questions and identify a single priority. Content experts acted as a resource to groups by identifying existing initiatives and literature to avoid duplication. Decisions on the prioritization of specific research questions remained in the hands of the PHU representatives (see Fig. 1 for an example of the outcome of the priority setting process).

**Table 2:** Phase 2 – Decision-making criteria

Criteria	Definition
Interest	Alignment with the priorities and direction of health units and the public health system
Impact	Ability to generate knowledge and evidence to support health units’ ability to meet the Ontario Public Health Standards and influence change in the public health system
Balance	Address the priorities of health units from different regions and of various sizes with the goal of meeting the demands of the majority and the needs of the minority

## Methods

After implementing two LDCP cycles, an evaluation was designed to ensure that the priority setting process was meeting its goals and using evidence-informed practices. No conceptual frameworks for evaluating research priority setting processes were identified through a review of academic and grey literature, although a plethora of articles were found describing a range of methods and various approaches used to select research priorities across disciplines and within specific contexts. After reviewing and assessing the literature for its applicability to the LDCP priority setting process, we chose the Checklist [11] to guide our evaluation. The Checklist articulates nine key elements that should be present within any research priority setting process to ensure a high quality exercise. We selected this framework because it captures the key constructs of a research priority setting exercise, its generic nature offers structure and flexibility, and it can be adapted to a variety of contexts. It was also expected that the Checklist would enable us to assess whether we were employing evidence-informed practices when conducting the LDCP research priority setting process, deciding on priorities, and translating the priorities into research. Specifically, the evaluation matrix shown in Table 3 outlines our adaptation of the Checklist to corresponding evaluation questions, indicators and data sources that guided the evaluation of the LDCP research priority setting process.

**Table 3:** ‘Nine Common Themes of Good Practice’ conceptual framework and its adaption for the evaluation of a research priority setting process

Theme	Description As outlined in the Checklist [11]	Evaluation questions Adapted from the Checklist [11]	Indicators Example of indicators used to inform analysis of LDCP research priority setting process	Data sources
Context	Articulating the contextual factors that underpin the process	1. Will the established goals, underlying values and principles continue to be relevant the next time the program facilitates priority setting? 2. Are there changes to the number of resources available for the next priority setting cycle?	i) Extent to which findings from original stakeholder engagement processes remain relevant for next research priority setting process ii) Availability of ongoing financial and human resources	i) Consultation with senior leadership within PHO ii) Program documents
Inclusiveness	Deciding who should be involved in setting research priorities	3. Did appropriate stakeholders participate in the most recent priority setting cycle, and was there balanced representation?	i) Number and representativeness of health units who submit phase 1 survey ii) Percentage of workshop participants who are front-line staff, managers, and senior decision-makers iii) Percentage of workshop participants who agree or strongly agree that they had opportunity to express opinions and ideas	i) Health unit demographic characteristics obtained from Phase 1 survey ii) Workshop registration list iii) Workshop evaluation iv) Informal feedback from program participants
Information gathering	Choosing what information should be gathered to inform the process	4. Was the most recent priority setting exercise appropriately informed? 5. Did the provided information sources support decision making?	i) Types of technical information provided to workshop participants ii) Perceived usefulness of technical material provided to workshop participants to aid decision-making	i) Workshop facilitation materials ii) Informal feedback
Planning for implementation	Establishing plans for translating research priorities into projects	6. In previous cycles, were there challenges to translating the research priorities into research?	i) Challenges reported relative to implementing LDCP project proposals ii) Quality and amount of support available from LDCP program staff	i) Interim and final progress reports ii) Consultation with program staff
Criteria	Selecting relevant criteria to focus discussion	7. In the most recent priority setting cycle, were the criteria effective for decision making, and will the criteria continue to be relevant for the next cycle?	i) Percentage of workshop participants who agree or strongly agree that the process helped them successfully apply the criteria ii) Alignment of criteria with overarching goals of research priority setting process	i) Workshop evaluation ii) Consultation with program advisory committee
Methods for deciding on priorities	Choosing a method for deciding on priorities	8. In the most recent priority setting cycle, were the methods for deciding on priorities appropriate and effective for decision making?	i) Percentage of workshop participants who agree or strongly agree that the process used to select priorities helped to build consensus ii) Percentage of workshop participants who agree or strongly agree that the workshop was an effective way to help health units discuss and prioritize topics for research and evaluation projects	i) Workshop evaluation
Use of a comprehensive approach	Assessing whether a comprehensive approach is necessary or if a tailored process and methods are required	9. Are there elements in comprehensive approaches and priority setting methods? Specifically, the Listening Model, COHRED, Child Health and Nutrition Research Initiative, Essential National Health Research, Combined approach matrix and Delphi technique, which are transferable to the LDCP priority setting process	i) Extent to which elements of established comprehensive approaches and methods can be transferred to or would strengthen LDCP priority setting processes	i) Review of comprehensive approaches and methods described in the Checklist
Transparency	Communicating the approach that was used to set priorities	10. Did all stakeholders receive information about the process and outcomes of the most recent priority setting process?	i) Types of communication strategies used to share information about the LDCP priority setting process with stakeholders ii) Timeliness of communications about the priority setting process	i) Program document review
Evaluation	Defining when and how evaluation of process and outcome will occur	11. Are further evaluation activities required to assess the delivery and outcomes of the priority setting process?	i) Perceived usefulness of current evaluation activities for informing quality improvements to the LDCP priority setting process	i) Consultation with program advisory committee

### Applying the Checklist to evaluate research priority setting

Evaluation questions were developed according to the nine themes described in the Checklist: context, inclusiveness, information gathering, planning for implementation, and use of a comprehensive approach, criteria, and methods for deciding on priorities, evaluation, and transparency. Next, indicators and data sources were identified and matched with evaluation questions (Table 3). A description of the LDCP priority setting process was prepared so that the goals, method and outcome of the process were clearly articulated for the analysis.

### Data analysis and interpretation

Data from program documents and informal feedback received from participants and staff were organized and mapped to the themes described in the Checklist, and evaluation questions were assessed by critically interpreting the existing evidence. Results from the workshop evaluation survey (completed by 59 participants for a 76% response rate) were also included. This survey assessed participants’ agreement or disagreement in having opportunities to express ideas and opinions (inclusiveness); whether the process helped participants to successfully apply the criteria of interest, impact and balance in decision-making (criteria); whether the process used to select priorities helped to build consensus (methods used to decide on priorities); and whether the workshop was an effective way to help PHUs discuss and prioritize topics for research and evaluation projects (methods used to decide on priorities).

The comprehensive approaches to priority setting and methods described in the Checklist were reviewed to inform recommendations to strengthen the research priority setting process. Finally, a consultation was held with a program advisory committee to ground the interpretation of results and recommendations. Program advisory committee members had extensive experience working with local PHUs and research institutions and, hence, were well positioned to offer contextual information integral to judging the appropriateness of proposed recommendations.

## Results

Using the Checklist as a framework to guide our evaluation resulted in several recommendations to improve elements of the LDCP research priority setting process.

### Theme 1 – Context

Since the program principles and goals had been recently established through a large stakeholder consultation exercise, no redefinition of the principles and goals was proposed. In addition, projected budget for the next priority setting exercise confirmed that similar funding and human resource supports would continue to be available. There were no recommended changes to the focus or scope of the priority setting exercise.

### Theme 2 – Inclusiveness

We examined the representativeness of PHU participation in the process, in alignment with the priority setting objective that decision-making for LDCP projects should be driven by PHUs. It was judged that PHUs were appropriately represented in both phases: 72% (26/36) of health units submitted a survey during Phase 1, and 78 participants from 28 of the 36 local PHUs attended the workshop (Phase 2). Balanced representation was assessed by examining the degree to which PHU front-line staff (e.g. nurses, epidemiologists and health promoters) and senior staff (e.g. managers and supervisors) participated in Phase 2 (workshop) of the process. Workshop registration information indicated that a range of PHU staff participated in the workshop. However, front-line PHU staff expressed a concern to PHO program staff that there was no mechanism for their ideas to be included if they were not selected by their health unit to attend the all-day workshop. In an effort to ensure the broadest and most inclusive representation possible, it was recommended that a new phase be integrated into the priority setting process allowing any PHU staff to submit an idea for a research or evaluation project within one of the prioritized subject areas in advance of the workshop. All ideas that were received would then move forward as an input to the workshop to be prioritized by workshop attendees.

### Theme 3 – Information gathering

Informal feedback received from workshop participants spoke of the need for technical data and contextual information related to each subject area to inform the dialogue and decision-making. Although having a content expert attend the workshop was seen as valuable for understanding whether particular research questions had already been addressed in the literature, workshop participants expressed a need to receive this information in advance. In response, it was recommended that evidence briefs be developed to highlight key gaps in the literature and that these briefs be provided to workshop participants in advance [17]. Having the information in advance would also allow for a more robust overview of the subject area, rather than participants being dependent on knowledge derived from one or two individuals.

### Theme 4 – Planning for implementation

Challenges associated with translating the prioritized research questions into feasible projects were identified. Several of the projects that were developed to answer the prioritized research questions had challenges meeting their objectives within the scope of the program’s timelines, and teams often lacked the knowledge and experience to effectively manage the implementation of the project. To build health unit capacity to develop more feasible research proposals, it was recommended that the time allocated for proposal development be extended, that additional educational workshops and training sessions be delivered by the LDCP program team, and that LDCP program staff provide greater support with project development and implementation. It was also recommended that the number of funded projects in the next program cycle be limited to allow for this increased emphasis on research capacity building, skills development and training.

### Theme 5 – Criteria

The majority of workshop participants (83%) agreed that the process guided their application of the criteria to their decision-making. The criteria were judged further by the program advisory committee for alignment with the established goals and objectives of priority setting. The balance and interest criterion aligned well with encouraging collaboration and partnership building between PHUs by requiring that priorities address the needs of multiple local PHUs. Additionally, it was determined that the impact criterion guided participants to select priorities that will generate knowledge that addresses a critical public health issue. No changes were recommended to the decision-making criteria.

### Theme 6 – Methods for deciding on priorities

The majority of workshop participants agreed that the nominal group technique used at the workshop helped build consensus (83%) and was effective in helping participants discuss and prioritise research and evaluation topics (88%). No changes were recommended to the methods for deciding on priorities.

### Theme 7 – Use of a comprehensive approach

Four existing comprehensive approaches identified in the Checklist (the COHRED management process to priority setting [18]; 3D Combined Approach Matrix [4]; Essential National Health Research approach [19]; The Child Health and Nutrition Research Initiative approach [20]) were considered in order to assess whether elements described in these established approaches were appropriate and transferable to the LDCP priority setting process. Of particular relevance to the LDCP process was the Essential National Health Research approach, which suggests using a taskforce with wide representation, such as researchers, policymakers and community representatives, to refine the agenda [19] and enhance consensus building. This supported the recommendation to increase balanced representation, as discussed under ‘inclusiveness’.

### Theme 8 – Transparency

At the start of each program cycle, the priority setting process was described in the program’s participation guidelines and an open webinar was held for the local public health audience, including information on how the criteria for priority setting were developed. Communication slide-decks provided information that could be readily shared with local PHU managers. In addition, after the completion of the cycle, a publically available written report was created that articulated the program goals, priority setting activities and outcomes of priority setting Additional file 1 [21]. Overall, it was determined that the types and timeliness of communication and dissemination activities in place were sufficient, and it was recommended that the program sustain its communication strategy.

### Theme 9 – Evaluation

The program advisory committee was consulted on whether additional evaluation activities were needed to inform improvements to the process. A decision was made to sustain the existing evaluation activities since these were found useful for assessing the themes in the Checklist. In addition, the program advisory committee recommended that short-term outcomes of priority setting, such as to what extent the set priorities were meeting LDCP program goals, be measured.

## Discussion

To our knowledge, this is the first paper to apply the Checklist as a conceptual framework to guide the evaluation of a research priority setting exercise. The application of the Checklist identified six recommendations to improve elements of the LDCP priority setting process: (1) introduction of a new component to the priority setting process to support balanced representation such that any PHU staff can submit an idea for a research or evaluation project in one of the prioritized subject areas (Theme 2 – inclusiveness); (2) the development and dissemination of evidence briefs to highlight key gaps in the literature to better inform decision-making (Theme 3 – information gathering); facilitating capacity for project implementation through (3) a reduction in the number of funded projects in the next cycle, (4) extended time allocated for project proposal development, and (5) greater emphasis on research capacity building, skills development and training (Theme 4 – planning for implementation); and (6) the evaluation of short-term outcomes of priority setting (Theme 9 – evaluation).

### Reflections on adapting and applying the Checklist

Our experience adapting and applying the Checklist suggests that this framework is suited to examining key constructs of a research priority setting process, and can provide guidance for developing research questions to frame an evaluation. However, the Checklist does not offer guidance on indicators or measurement tools that can be utilized. The current analysis primarily used document review and routinely collected information sources to evaluate the nine priority setting themes, and assumes this is a valid application of the Checklist. Further guidance on appropriate evaluation methods and indicators could provide greater support for evaluation and application of the Checklist within research priority setting.

The Checklist places the theme ‘review of a comprehensive approach’ as a preparatory step in developing and planning a research priority setting process. We chose to move this theme toward the end of the evaluation process. The comprehensive approaches identified by the Checklist were not directly applicable to our context as our evaluation focused on strengthening our established process rather than adopting an entirely new approach. In our adaptation, we chose to utilize this theme as an opportunity to inform our recommendations. We suggest that others also utilize this theme to reflect on potential strategies from established approaches that are applicable and transferable to their settings to ensure that the process is as comprehensive and complete as possible.

Applying the Checklist for our evaluation revealed that ‘timing’ was an underlying factor among several themes. For the ‘inclusiveness’ theme, it was useful to consider whether the time at which different stakeholders were invited to the process was conducive to setting priorities. Similarly, examining ‘information gathering’ practices indicated at which time intervals information sources would be most facilitative to decision-making. Furthermore, considering whether an appropriate amount of time was allocated to different elements of the process, such as implementation planning, provided guidance for re-structuring the timeline of proposal development in order to enhance project implementation. The findings suggest that ‘timing’ is an important indicator to consider in the evaluation of a research priority setting process, and could be incorporated into multiple evaluation questions specific to research priority setting themes. We suggest incorporating this concept into the Checklist.

The feasibility of the LDCP program to act on the recommendations identified through the adaptation and application of the Checklist was aided by the degree of control over the process. To illustrate, the LDCP program facilitates both the research priority setting and project implementation phases. PHU representatives set and implement the research priorities with funding provided by the LDCP program. As LDCP research priority setting and implementation are closely aligned and managed by the same organization, recommendations to strengthen the process could be readily addressed. Resource constraints and lack of support among different stakeholders on research priorities have been identified as barriers to research implementation in previously reported priority setting exercises [22, 23] and perhaps reflects the need to establish mechanisms that can better align research priority setting and implementation. Indeed, plans for implementation have been under-reported among national priority setting exercises [24, 25].

The Checklist emphasizes that a priority setting process should strive for a comprehensive and engaged approach. Engagement takes energy and commitment from those involved, and it can be challenging to deliver a process that will achieve all ‘nine themes of good practice’ within the resources available. In line with the Checklist’s vision of tailoring exercises to suit the capacity and constraints of a particular context, our findings illustrate that evaluation can assist with refining a priority setting process so that it contains the best suited combination of elements needed to effectively meet the context and objectives of the priority setting exercise.

### Limitations

The application of the Checklist to an evaluation of a research priority setting process has some limitations. The Checklist was designed for planning such a process, and its application as an evaluation tool has not been validated. Nonetheless, the Checklist provides themes and definitions for constructs that should be present in any priority setting exercise, offering flexibility to apply the themes to evaluation questions and to tailor indicators to the context of the research priority setting exercise. It was assumed that the adapted questions we used represent the constructs as intended. In addition, we had a reduced ability to comprehensively assess all ‘nine themes’ since the evaluation was informed, in part, by a secondary analysis of information sources that were not collected for the purposes of this evaluation. The design of measurement tools that can assess the constructs of the Checklist can be considered in future evaluations.

## Conclusion

Applying the Checklist and its themes to the LDCP research priority setting process led to a series of recommendations and reinforced key aspects of the process. Overall, the evaluation confirmed that the LDCP priority setting process is meeting its goals and is employing an evidence-informed approach for setting research priorities.

There are few published evaluations of research priority setting processes reported in the literature [24]. Yet, evaluation is fundamental to the selection of relevant research priorities. This paper offers direction on how one might apply the Checklist and its themes to the evaluation of a research priority setting exercise. The Checklist is particularly useful for assessing whether the priority setting process is achieving key constructs relevant to planning the process, deciding on priorities and post-priority setting work. Ultimately, the implementation of an effective research priority setting process will facilitate the allocation of resources to research priorities that are most important in a given context.

## Additional file

Additional file 1: 2012 LDCP Workshop 1 Final Report: Moving from possibilities to projects. (PDF 980 kb)
