# Supplementary material for: Using the Nine Common Themes of Good Practice checklist as a tool for evaluating the research priority setting process of a provincial research and program evaluation program
Source: Health Res Policy Syst. 2016 Mar 23;14:22. doi: 10.1186/s12961-016-0092-5 (PMC4804477; doi:10.1186/s12961-016-0092-5)
Supplement: Additional file 1: — 2012 LDCP Workshop 1 Final Report: Moving from possibilities to projects. (PDF 980 kb) [file 12961_2016_92_MOESM1_ESM.pdf]

2012 LDCP Workshop 1: Moving from Possibilities to Projects

Workshop 1 Report

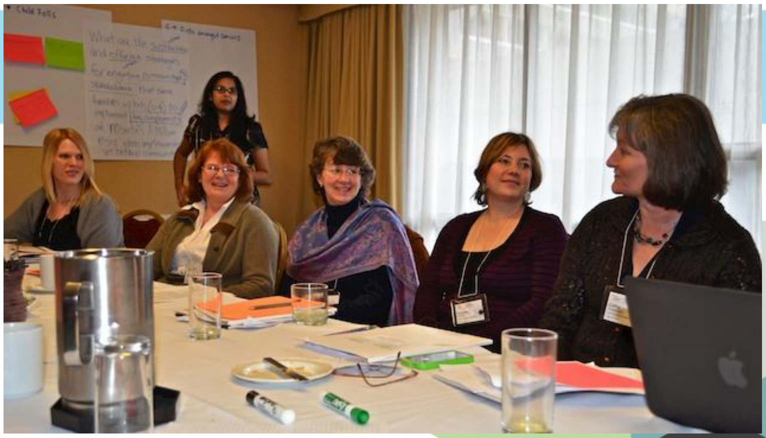

March 30, 2012

Public Health Ontario
PARTNERS FOR HEALTH

Santé publique Ontario
PARTENAIRES POUR LA SANTÉ

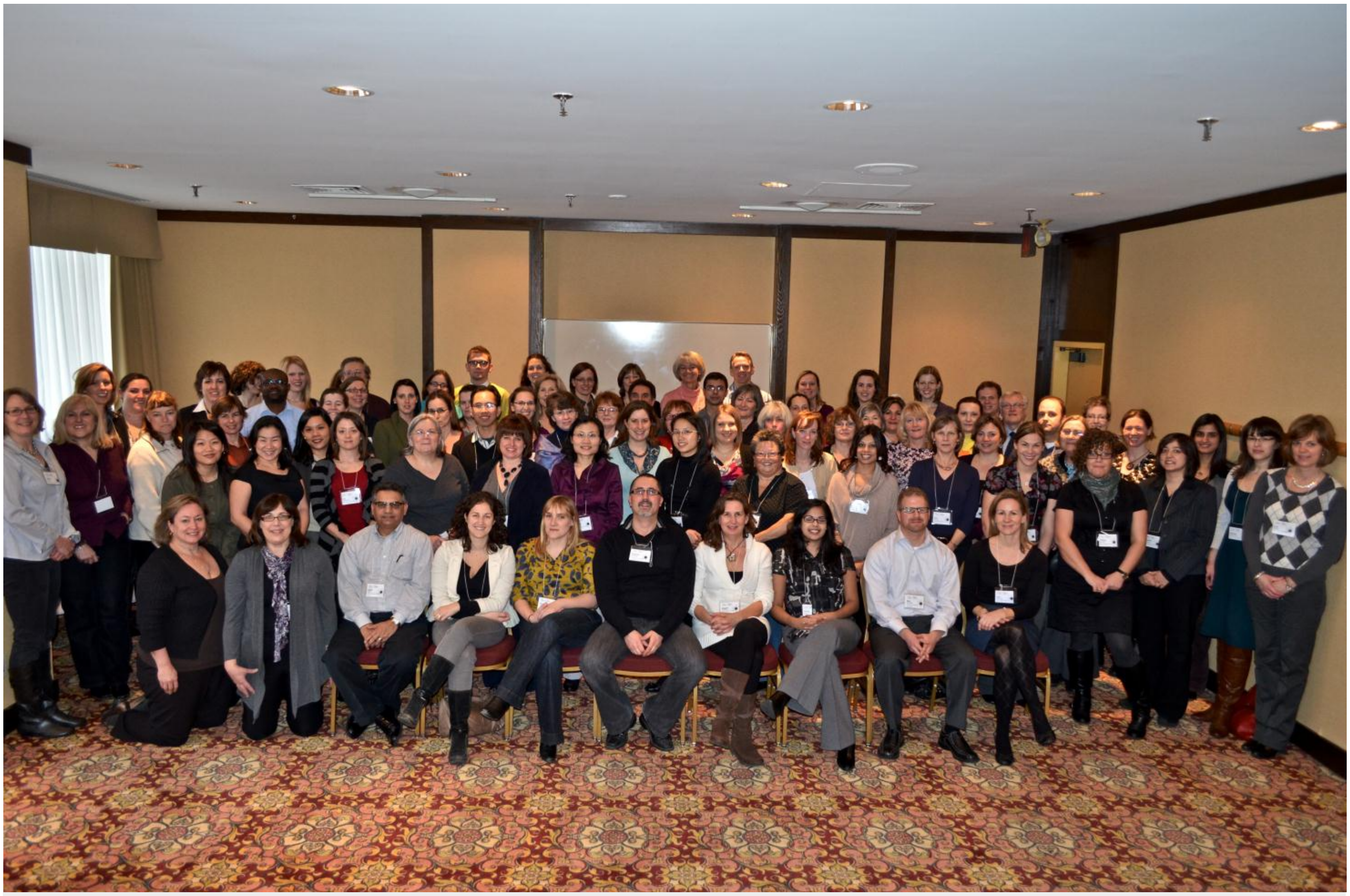

2012 LDCP Workshop 1 Participants
Photo credits: Claude Martel/Public Health Ontario

1.0 Introduction

On March 2nd, 2012, Public Health Ontario facilitated *Moving from Possibilities to Projects*, the first workshop in the 2012 Locally-Driven Collaborative Projects (LDCP) program. Seventy-eight representatives from 27 public health units across Ontario, as well as 13 content experts, attended the session. *Moving from Possibilities to Projects* is the first in a three-part series of workshops that, together, are designed to assist health units to build partnerships and jointly develop a research or evaluation proposal that addresses a critical public health need.

The objectives of Workshop 1 were to:

1. Identify and discuss potential project topics within pre-registered Ontario Public Health Standards (OPHS) Program Standards subject areas;
2. Allow health unit staff to prioritize topics by applying the criteria of impact, interest, and balance; and,
3. Begin to form partnerships for collaboration.

Public Health Ontario designed a series of activities to assist subject area groups to start to establish collaborative teams, develop "just right" research and evaluation questions, and explore the extent to which each question met the priority setting criteria. The purpose of this report is to summarize the main activities of the workshop, and describe the process and results of the priority setting activity. For a broad overview of the day's activities, please see Appendix A for a workshop agenda. The results of the workshop evaluation are available in Appendix B.

2.0 Priority Setting Process

Participants were asked to register for one of seven different subject areas (Table 1) that were selected through the Research and Evaluation Topic Survey that was administered to public health units in January 2012.

Each subject area group had representation from a range of public health units and was provided with the list of topics generated through the Research and Evaluation Topic Survey within their subject area. Over the course of the day, subject area teams, led by a facilitator, worked towards the prioritization of a single research or evaluation question that will form the basis of their collaborative project.

TABLE 1. SUBJECT AREAS IDENTIFIED AS PRIORITIES BY HEALTH UNITS FOR 2012
Vaccine Preventable Diseases - Immunization
Falls Across the Lifespan
Built Environment
Breastfeeding
Alcohol and Other Substances
Healthy Pregnancies
Social Media and Technology

At different stages, participants were asked to consider each of the following three priority setting criteria as they discussed and selected their top choices (see Figure 1):

Interest: Alignment with the priorities and direction of health units and the public health system.

Impact: Ability to generate knowledge and evidence, to support health units to meet the Ontario Public Health Standards and influence change in the public health system.

Balance: Address the priorities of health units from different regions and of various sizes with the goal of meeting the demands of the majority and the needs of the minority.

Content experts from a range of organizations – including Public Health Ontario, the Alcohol Policy Network, the Ontario Neurotrauma Foundation, SMARTRISK, and the University of Toronto – acted as a resource to the LDCP collaborative teams by helping to identify existing initiatives and providing methodological support. Although content experts provided invaluable input into the discussions, decisions surrounding the prioritization of specific research questions remained in the hands of the health units.

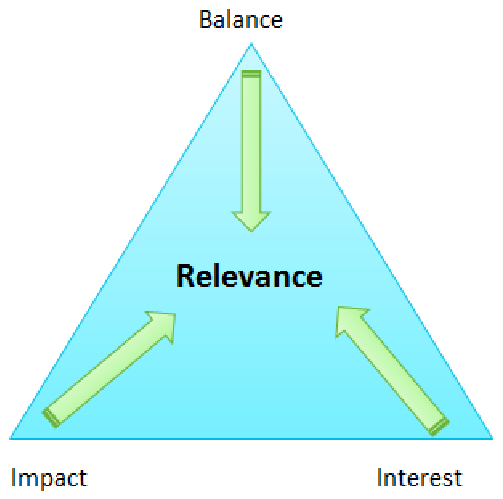

Figure 1. Priority setting criteria used to identify top research or evaluation question.

3.0 Workshop Outcomes

Below is a brief overview of the outcomes of Workshop 1. Each table includes the top five research or evaluation questions that were developed through a group consensus and voting process, and highlights in blue the final question that will form the basis of each LDCP subject area's ongoing collaboration. N/A refers to questions that were not among the LDCP collaborative team's top 3 choices and, consequently, were not ranked. For a list of participants in each subject area group, please see Appendix C.

3.1 VACCINE PREVENTABLE DISEASES – IMMUNIZATION

TOP 5 RESEARCH OR EVALUATION QUESTIONS: VACCINE PREVENTABLE DISEASES - IMMUNIZATION

	FINAL RANK
**What parent/school-based strategies are effective to increase immunization coverage rates in school-aged children?**	**1**
What is the feasibility of connecting existing data (OHIP billing, FHT, IRIS, other) to a provincial immunization strategy?	**2**
What are primary factors that influence administration of school-based vaccines? (E.g. system barriers, inadequate HR, failure to obtain consents, age at which immunization is given, perceived risk of disease vs. risk of immunization).	**3**
What strategies targeted at parents/guardians/students are effective to increase uptake of HPV vaccines?	**N/A**
What factors influence the public's acceptance of various vaccines? (E.g. HPV, influenza)	**N/A**

3.2 FALLS ACROSS THE LIFESPAN

TOP 5 RESEARCH OR EVALUATION QUESTIONS: FALLS ACROSS THE LIFESPAN

	FINAL RANK
**What are the sustainable and effective strategies for engaging community stakeholders that serve families with children 0-4 years to implement key components of Alberta's Million Message program when adopted and implemented in Ontario communities?**	**1**
What are the best practices to prevent sport and recreational injury among children 10-19 in Ontario?	**2**
How might falls prevention efforts be translated through the lifespan to maximize impact at every age?	**3**
What are the perceived barriers and effective strategies for delivering consistent and repetitive messaging among stakeholders that service families with children ages 0-4 years?	**N/A**
What are the best practices for preventing future adult falls to inform the development of a strategy for Ontario?	**N/A**

3.3 BUILT ENVIRONMENT

TOP 5 RESEARCH OR EVALUATION QUESTIONS: BUILT ENVIRONMENT	FINAL RANK
**What are the best practices, tools and suitable alternatives in planning policies and procedures for rural and small towns for improving health outcomes within the built environment?**	**1**
What are the barriers to implementing set-backs to protect sensitive populations from environmental exposures?	**2**
What are the economic indicators and health benefits that demonstrate the value of active transportation and transportation policy?	**3**
What are the barriers and facilitations to using an economic argument for active transportation to influence policy at the municipal and provincial level?	**N/A**
What is the economic impact (including health and localization of healthy food systems) of land use planning?	**N/A**

3.4 BREASTFEEDING

TOP 4 RESEARCH OR EVALUATION QUESTIONS: BREASTFEEDING	FINAL RANK
**What is the feasibility of public health units in Ontario establishing a common infant feeding surveillance system that will collect comparable, ongoing data on breastfeeding?**[1]	**1**
What survey questions and tools exist that are reliable for calculating desired breastfeeding indicators?	**N/A**
How can Public Health Units in Ontario establish an infant feeding surveillance system that will collect ongoing data on breastfeeding?	**N/A**
What are the evidence-informed strategies for increasing breastfeeding intentions, initiation, duration, and exclusivity?	**N/A**
What are the challenges, barriers, and steps to overcome implementing the 10 steps of BFI and recommendations for overcoming barriers?	**N/A**

[1] The final research question selected to move forward was an amalgamation of the two questions below it in the table.

3.5 ALCOHOL AND OTHER SUBSTANCES

TOP 5 RESEARCH OR EVALUATION QUESTIONS: ALCOHOL AND OTHER SUBSTANCES

	FINAL RANK
**What are the supports required to design and implement a coordinated comprehensive strategy applied to the local level to reduce drinking in excess of the Low-Risk Drinking Guidelines?**	**1**
What are the barriers and facilitators to implementing screening and brief interventions for HCPs?	**2**
What supports are required to design and implement a coordinated social marketing/brief intervention strategy to reduce drinking in excess of LRDG?	**N/A**
What are the best practices for implementing alcohol warning labels to encourage within the LRDG?	**N/A**
What is the relationship between alcohol outlet density and high risk drinking in Ontario communities?	**N/A**

3.6 HEALTHY PREGNANCIES

TOP 5 RESEARCH OR EVALUATION QUESTIONS: HEALTHY PREGNANCIES

	FINAL RANK
**An evaluation of the effectiveness of online prenatal programs amongst reproductive-aged population in Ontario**	**1**
To assess use of BORN information system for population health surveillance in following PH priorities: 1. Alcohol use, illicit drug use during pregnancy. 2. FASD. 3. Maternal weight gain	**2**
What is the current rate of pregnant women having a discussion with HCP re: importance of physical activity and healthy weights in pre-conception and pregnancy? (What strategies can HU implement to increase rate)	**3**
To evaluate the effectiveness of the use of social media tools to increase reach and awareness related to prenatal health	**N/A**
What are the determinants/SDOH factors resulting in high rates of teen pregnancy in rural and or northern Ontario	**N/A**

3.7 SOCIAL MEDIA AND TECHNOLOGY

TOP 5 RESEARCH OR EVALUATION QUESTIONS: SOCIAL MEDIA AND TECHNOLOGY	FINAL RANK
**How can existing evidence on the effectiveness of social media translate to public health practice as a part of comprehensive programs and strategies at health units in Ontario?**	**1**
How can existing evidence on the effectiveness of SM translate to PH practices as part of programs and strategies to advance health public policy?	**2**
How can existing evidence on the effectiveness of SM translate to PH practice as part of programs and strategies to enhance client engagement?	**3**
How can existing evidence on SM effectiveness translate to PH practices as part of programs and strategies to influence personal health practices?	**N/A**
To determine/identify best practices for using social media for client engagement.	**N/A**

4.0 Next Steps

The second of three workshops, *Building Collaborative Teams, Beginning Proposals,* will be held on April 5th, 2012 and April 13th, 2012. Public Health Ontario is busy planning a range of activities that will assist LDCP collaborative teams to further scope their research or evaluation questions, develop SMART objectives and begin discussing team member roles and responsibilities. Throughout the day, LDCP teams will also learn more about library services as well as other resources and supports that are available from PHO.

The objectives of Workshop 2 are to:

1. Work in collaborative teams to develop proposals including research objectives and methods
2. Introduce participants to tools and resources available to support proposal development
3. Complete an action plan, identifying roles and responsibilities of team members

Similar to Workshop 1, context experts will be available to provide methodological support and insight into current projects and initiatives that are happening provincially and nationally. Representatives from community organizations and associations that are interested in partnering or collaborating on the LDCP projects will also be in attendance.

In order to support LDCP 2012 teams as they work on their proposals, Public Health Ontario will host Workshop 3 on May 31st, 2012. Workshop 3 will provide LDCP collaborative teams with an opportunity to come together to develop their proposals. The focus of Workshop 3 will be on assisting teams with specific aspects of their project planning, such as developing timelines and Knowledge Exchange plans, and budgeting. LDCP proposals are due on July 13, 2012. More information about 2012 LDCP important dates can be found on PHO`s website.

Appendix A. Workshop Agenda

2012 LDCP Workshop 1 Agenda

FRIDAY, MARCH 2, 2012 - 08:30AM TO 16:30PM

DELTA CHELSEA HOTEL, TORONTO

TIME	PRESENTER	TOPIC	ROOM
**08:30**		Registration and Networking Breakfast	Rosseti Room
**09:00**	Brenda Mitchell	Welcome	Rosseti Room
**09:15**	Vinita Haroun	2012 LDCP Workshop 1 Overview	Rosseti Room
**09:35**	Sheila Cook	Model of Collaborative Work and Decision-Making Styles	Rosseti Room
**09:55**		Break	Rosseti Room
**10:10**	Subject Area Facilitators	Priority Setting Session A: Steps 1 to 5	Breakout Rooms
**12:30**		Lunch	Rosseti Room
**13:15**	Sheila Cook	"The Dip"	Rosseti Room
**13:30**	Cathy Dykeman	"Tips and Tricks from the Trenches: Elements of a Successful Collaboration"	Rosseti Room
**13:45**	Subject Area Facilitators	Priority Setting Session B: Steps 6 and 7	Breakout Rooms
**15:00**		Break and Project Tour	Rosseti Room
**15:20**	Lea Narciso	Next Steps	Rosseti Room
**15:45**	Subject Area Facilitators	Action Planning Session C: Step 8	Rosseti Room
**16:15**	Vinita Haroun	Wrap Up and Evaluation	Rosseti Room

Appendix B. Workshop Evaluation

At the end of the workshop, participants were asked to complete a short survey that explored the extent to which they felt that Workshop 1 met its stated objectives. Fifty-nine participants completed the questionnaire, a response rate of 64%.

WORKSHOP SUCCESSES

Participants noted that there were a range of excellent components to Workshop 1. In particular, participants commented on the collaborative nature of the day and the *"opportunity to articulate common areas of interest [and] potential areas for effort."* Several participants also noted that the facilitators were *"great"* and valued the "*guidance by PHO."*

These comments were reflected in the average responses to the five structured questions included in the evaluation form. Figure 2 displays the percentage of participants that agreed or disagreed with each of the five structured statements in the questionnaire based on a scale where 1 indicated strongly disagree and 5 indicated strongly agree.

FIGURE 2. PARTICIPANT RESPONSES TO THE EVALUATION SURVEY

THE COLLABORATION WORKSHOP WAS AN EFFECTIVE WAY TO LET HEALTH UNITS DISCUSS AND PRIORITIZE TOPICS FOR RESEARCH AND EVALUATION PROJECTS

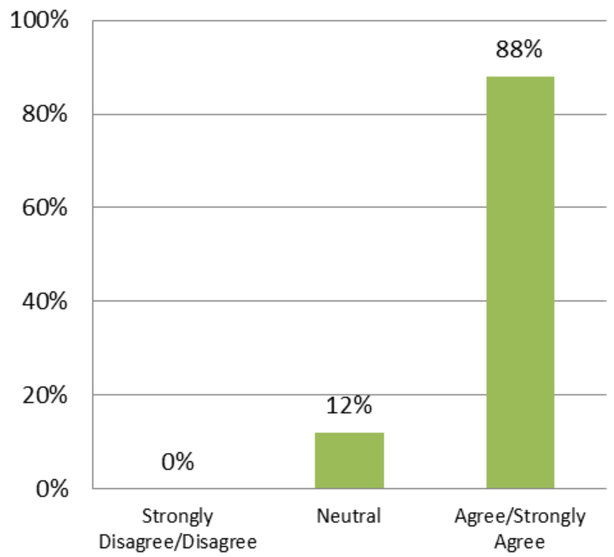

I FELT THAT THE PROCESS USED THIS YEAR HELPED PARTICIPANTSTO SUCCESSFULLY APPLY THE CRITERIA OF INTEREST, IMPACT AND BALANCE IN DECISION-MAKING.

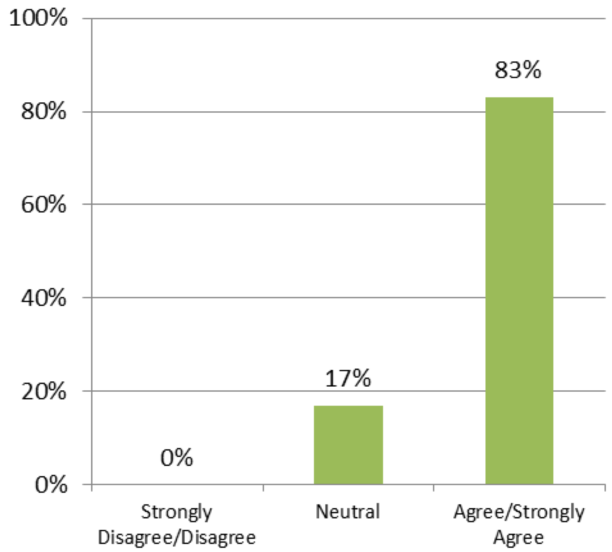

I FELT THE WORKSHOP WAS A GOOD FIRST STEP TO BUIDLING EFFECTIVE PARTNERSHIPS AND COLLABORATIONS WITH OTHERS WHO ARE INTERESTED IN RESEARCH AND EVALUATION IN PUBLIC HEALTH

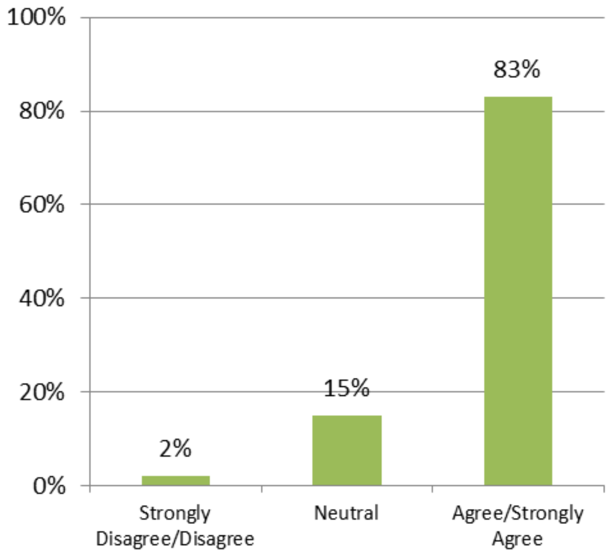

I FELT THAT THE PROCESS USED TO SELECT PRIORITIES HELPED TO BUILD CONSENSUS

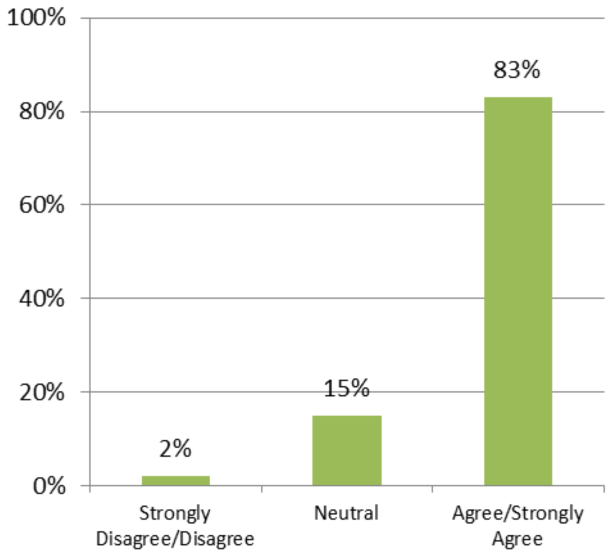

I HAD OPPORTUNITIES TO EXPRESS MY OPINIONS AND IDEAS

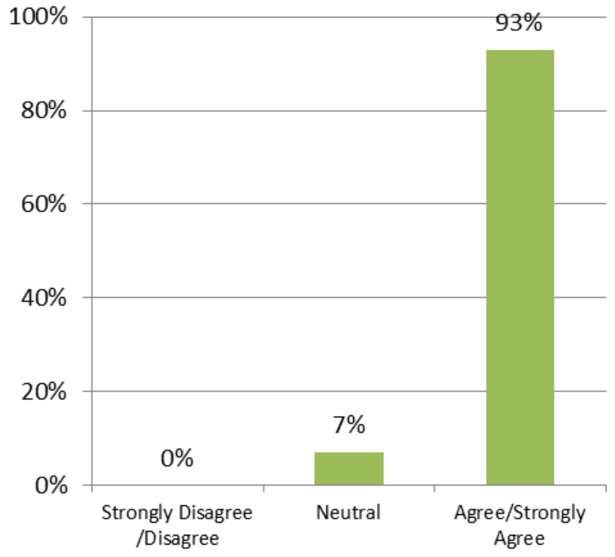

RECOMMENDATIONS

While there were many positive comments about Workshop 1, participants also provided several recommendations for future workshops. Participants would like to have "*more nutritious food*" and to ensure that the day's activities remain on time. In terms of the priority setting process, some participants wanted *"more information on how to formulate research questions"* and the opportunity to discuss and debrief after a voting process had occurred.

Appendix C. Participant List

NAME	JOB TITLE	ORGANIZATION	SUBJECT AREA
Jayne Caldwell	Policy Analyst	Toronto Drug Strategy Secretariat, Toronto Public Health	Alcohol and Other Substances
Amanda Chan	Public Health Nurse	York Region Public Health Services	Alcohol and Other Substances
Adrienne Chin	Health Promotion Consultant	Toronto Public Health	Alcohol and Other Substances
Bernadette Garrity	Public Health Nurse	Middlesex-London Health Unit	Alcohol and Other Substances
Melissa Hutchinson	Program Manager, Public Health	Durham Region Health Department	Alcohol and Other Substances
Amanda Kroger	Manager	Region of Waterloo Public Health	Alcohol and Other Substances
Novella Martinello	Foundational Standard Specialist	Kingston, Frontenac and Lennox & Addington Public Health	Alcohol and Other Substances
Sherri Preszcator	Public Health Manager	Huron County Health Unit	Alcohol and Other Substances
Melissa Rennison	Public Health Nurse	Middlsex-London Health Unit	Alcohol and Other Substances
Michelle Schwarz	Public Health Nurse	Halton Region Health Department	Alcohol and Other Substances
Vanessa Trumpickas	Health Promotion Specialist	Hamilton Public Health Services	Alcohol and Other Substances
Janice Bogdan	Program Manager	Durham Region Health Department	Breastfeeding
Shelly Brown	Family Health Program Manager	Hastings & Prince Edward Counties Health Unit	Breastfeeding
Eileen Chuey	Public Health Nurse	Halton Region Health Department	Breastfeeding
Deanna Cole-Benjamin	Public Health Nurse	Kingston, Frontenac and Lennox & Addington Public Health	Breastfeeding
Sandy Dupuis	Epidemiologist	Niagara Region Public Health Department	Breastfeeding
Shanna Hoetmer	Epidemiologist	York Region Public Health Services	Breastfeeding
Sharmin Jaffer	Manager, Child and Family Health Promotion	Region of Waterloo Public Health	Breastfeeding
Heather Lokko	Manager, Reproductive Health Team	Middlesex-London Health Unit	Breastfeeding
Ronda Manning	Supervisor, Health Promotion	Oxford County Public Health & Emergency Services	Breastfeeding

NAME	JOB TITLE	ORGANIZATION	SUBJECT AREA
Debbie Silvester	Manager, Family Health	Windsor-Essex County Health Unit	Breastfeeding
Renée St Onge	Manager, Research and Evaluation	Sudbury & District Health Unit	Breastfeeding
Janet Vandenberg	Public Health Nurse	York Region Public Health Services	Breastfeeding
Fangli Xie	Epidemiologist	Durham Region Health Department	Breastfeeding
Erica Arnett	Health Promoter	Elgin-St. Thomas Health Unit	Built Environment
Fabio Cabarcas	Senior Policy Analyst	Halton Region Health Department	Built Environment
Carolyn Coppens	Epidemiologist	Elgin-St. Thomas Health Unit	Built Environment
Helen Doyle	Manager, Environmental	York Region Public Health Services	Built Environment
Jackie Gervais	Health Promoter	Niagara Region Public Health Department – CDIP	Built Environment
Lisa Kaldeway	Health Promoter	Haliburton, Kawartha, Pine Ridge District Health Unit	Built Environment
Iqbal Kalsi	Manager, Health Hazards, Environmental Health	Middlesex-London Health Unit	Built Environment
Alanna Leffley	Senior Epidemiologist	Grey Bruce Health Unit	Built Environment
Karen Loney	Health Promoter	Chatham-Kent Health Unit	Built Environment
Donna Taylor	Director, Health Protection	Perth District Health Unit	Built Environment
Deanna White	Epidemiologist	Haldimand-Norfolk Health Unit	Built Environment
Shawn Zentner	Manager, Health Protection	Wellington-Dufferin-Guelph Health Unit	Built Environment
Nicole Dupuis	Program Manager	Chatham-Kent Health Unit	Built Environment
Loretta Bernard	Manager - Injury & Substance Misuse Prevention	York Region Public Health Services	Falls Across the Lifespan
Cindy Kirkpatrick	Public Health Nurse	Hastings & Prince Edward Counties Health Unit	Falls Across the Lifespan
Etta Li	Public Health Nurse	York Region Public Health Services	Falls Across the Lifespan
Casey Walters Gray	Public Health Nurse	Kingston, Frontenac and Lennox & Addington Public Health	Falls Across the Lifespan
Jacqueline Whittingham	Manager, Chronic Disease & Injury Prevention	Hastings & Prince Edward Counties Health Unit	Falls Across the Lifespan

NAME	JOB TITLE	ORGANIZATION	SUBJECT AREA
Leslie Wright	Public Health Nurse	Algoma Public Health	Falls Across the Lifespan
Carolyn Wilkie	Public Health Nurse	Halton Region Health Department	Falls Across the Lifespan
Melissa Horan	Health Promotion Specialist	Wellington-Dufferin-Guelph Health Unit	Healthy Pregnancies
Zahra Kassam	Manager, Reproductive Health	York Region Public Health Services	Healthy Pregnancies
Melissa Lonnee	Public Health Nurse	Middlesex-London Health Unit	Healthy Pregnancies
Sue Morris	Manager, Reproductive Health	Region of Waterloo Public Health	Healthy Pregnancies
Fidel Obu	Public Health Epidemiologist	Porcupine Health Unit	Healthy Pregnancies
Kate O'Connor	Director, Research and Evaluation	Kingston, Frontenac and Lennox & Addington Public Health	Healthy Pregnancies
Samantha Petkou	Public Health Nurse	York Region Public Health Services	Healthy Pregnancies
Mary-Anne Pietrusiak	Epidemiologist	Durham Region Health Department	Healthy Pregnancies
Jennifer Schmidt	Reproductive Health Supervisor	Halton Region Health Department	Healthy Pregnancies
Lori Webel-Edgar	Program Manager	Simcoe Muskoka District Health Unit	Healthy Pregnancies
Carmen Yue	Epidemiologist	Toronto Public Health	Healthy Pregnancies
Shannon Aitchison	Public Health Nurse	Middlesex-London Health Unit	Social Media and Technology
Roberto Almeida	Program Manager	Hastings & Prince Edward Counties Health Unit	Social Media and Technology
Lois Bailey	Chief Financial Officer	Northwestern Health Unit	Social Media and Technology
Cate Bannan	Manager, Sexual Health and Needle Exchange Program	Halton Region Health Department	Social Media and Technology
Carrie Beatty	Communications Specialist	Niagara Region Public Health Department	Social Media and Technology
Chris Bowes	Research and Policy Analyst	North Bay Parry Sound District Health Unit	Social Media and Technology
Nancy Gan	Public Health Educator	York Region Public Health Services	Social Media and Technology
Duc Mai	Public Health Inspector	Middlesex-London Health Unit	Social Media and Technology
Heather McCully	Project Manager, Social Media	City of Hamilton Public Health & Community Services Department	Social Media and Technology

NAME	JOB TITLE	ORGANIZATION	SUBJECT AREA
Kris Millan	Manager, CQI and Central Resources	Kingston, Frontenac and Lennox & Addington Public Health	Social Media and Technology
Anne Schlorff	Director, Central Resources	Region of Waterloo Public Health	Social Media and Technology
Megan Williams	Health Promotion Specialist	Simcoe Muskoka District Health Unit	Social Media and Technology
Joanne Cameron	Manager, VPD	Toronto Public Health	Vaccine Preventable Diseases - Immunization
Robin Cooper	Health Promotion Planner	Thunder Bay District Health Unit	Vaccine Preventable Diseases - Immunization
Jennifer Duffin	Public Health Manager	Perth District Health Unit	Vaccine Preventable Diseases - Immunization
Ian Gemmill	Medical Officer of Health	Kingston, Frontenac and Lennox & Addington Public Health	Vaccine Preventable Diseases - Immunization
Lorraine Grypstra	Public Health Nurse	City of Hamilton Public Health & Community Services Department	Vaccine Preventable Diseases - Immunization
Chris Harold	Manager, Information and Planning	Region of Waterloo Public Health	Vaccine Preventable Diseases - Immunization
Kathy Jovanovic	Acting Manager of Communicable Disease Control	Halton Region Health Department	Vaccine Preventable Diseases - Immunization
Ameeta Mathur	Manager, VPD	Toronto Public Health	Vaccine Preventable Diseases - Immunization
Carly McKinnon	Assistant Manager	Durham Region Health Department	Vaccine Preventable Diseases - Immunization
Peggy Patterson	Coordinator, Program Planning and Evaluation	Renfrew County and District Health Unit	Vaccine Preventable Diseases - Immunization
Michelle Piett	Team Leader, VPD	Niagara Region Public Health Department	Vaccine Preventable Diseases - Immunization
